# What makes a molecule a pre‐ or a post‐herbicide – how valuable are physicochemical parameters for their design?

**DOI:** 10.1002/ps.6535

**Published:** 2021-07-19

**Authors:** Hansjörg Krähmer, Helmut Walter, Peter Jeschke, Klaus Haaf, Peter Baur, Richard Evans

**Affiliations:** ^1^ Kantstrasse 20 D‐65719 Hofheim Germany; ^2^ AgroField Consulting Obrigheim Germany; ^3^ Research & Development, Crop Science, Pest Control Chemistry Bayer AG Monheim am Rhein Germany; ^4^ Research & Development, Crop Science, Weed Control Chemistry Bayer AG Frankfurt am Main Germany; ^5^ CropPromotion Advice Schondorf am Ammersee Germany; ^6^ Richard Evans Raleigh NC USA

**Keywords:** root uptake, translocation, application timing, physicochemical parameters, site of action, weed spectrum

## Abstract

Pre‐emergence herbicides are taken up by seeds before germination and by roots, hypocotyls, cotyledons, coleoptiles or leaves before emergence, whereas post‐emergence herbicides are taken up primarily by foliage and stems. Most modern pre‐emergence herbicides are lipophilic, but post‐emergence herbicides may be lipophilic or hydrophilic. The metabolic conversion of herbicides to inactive or active metabolites after plant uptake is of major importance for some compound classes. Several herbicides are proherbicides as for example some acetyl‐coenzyme A carboxylase (ACCase)‐inhibitors. The physicochemical characteristics of proherbicides and herbicides are usually unrelated. A major role can be attributed to the site of action at a cellular level. A great number of herbicides such as photosystem II (PS II)‐inhibitors, protoporphyrinogen oxidase (PPO)‐inhibitors or carotenoid biosynthesis inhibitors require light for activity. Others, such as cellulose‐biosynthesis and mitotic inhibitors seem to be primarily active in belowground organs. Several lipophilic barriers against the uptake of xenobiotics exist in aboveground and belowground plant parts. The relevance of these barriers needs, however, further clarification. Uptake and translocation models are valuable tools for the explanation of the potential movement of compounds. Many factors other than uptake and translocation have, however, to be considered for the design of herbicides. For post‐emergence herbicides, ultraviolet (UV) light stability, stability in formulations, and mixability with other agrochemicals have to be kept in mind while, in addition to the aforementioned factors soil interaction plays a major role for pre‐emergence herbicides. In our opinion, general physicochemical characteristics of pre‐ or post‐emergence herbicides do, unfortunately not exist yet. © 2021 The Authors. *Pest Management Science* published by John Wiley & Sons Ltd on behalf of Society of Chemical Industry.

## Graphical abstract

A high number of parameters influence the fate of herbicides. General characteristics of pre‐ or post‐emergence herbicides can therefore not be defined yet.

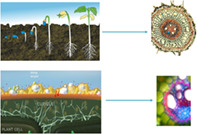

## 1 INTRODUCTION

Modern weed management involves many different tools such as the use of competitive crop varieties, cultural/mechanical, biological and chemical weed control.

Herbicides can be applied before planting, after planting or post‐harvest. Some herbicides are incorporated into the soil to guarantee a better performance, while others are applied banded in rows only or post‐directed using special nozzles. We concentrate here on planted, arable crops and analyze pre‐emergence and post‐emergence herbicides in row crops other than rice. Flooding of rice fields with water for example leads to the requirement of products with special characteristics. Weed spectra in rice and crop management are different compared with other field crops.^1^

**Figure 1:**
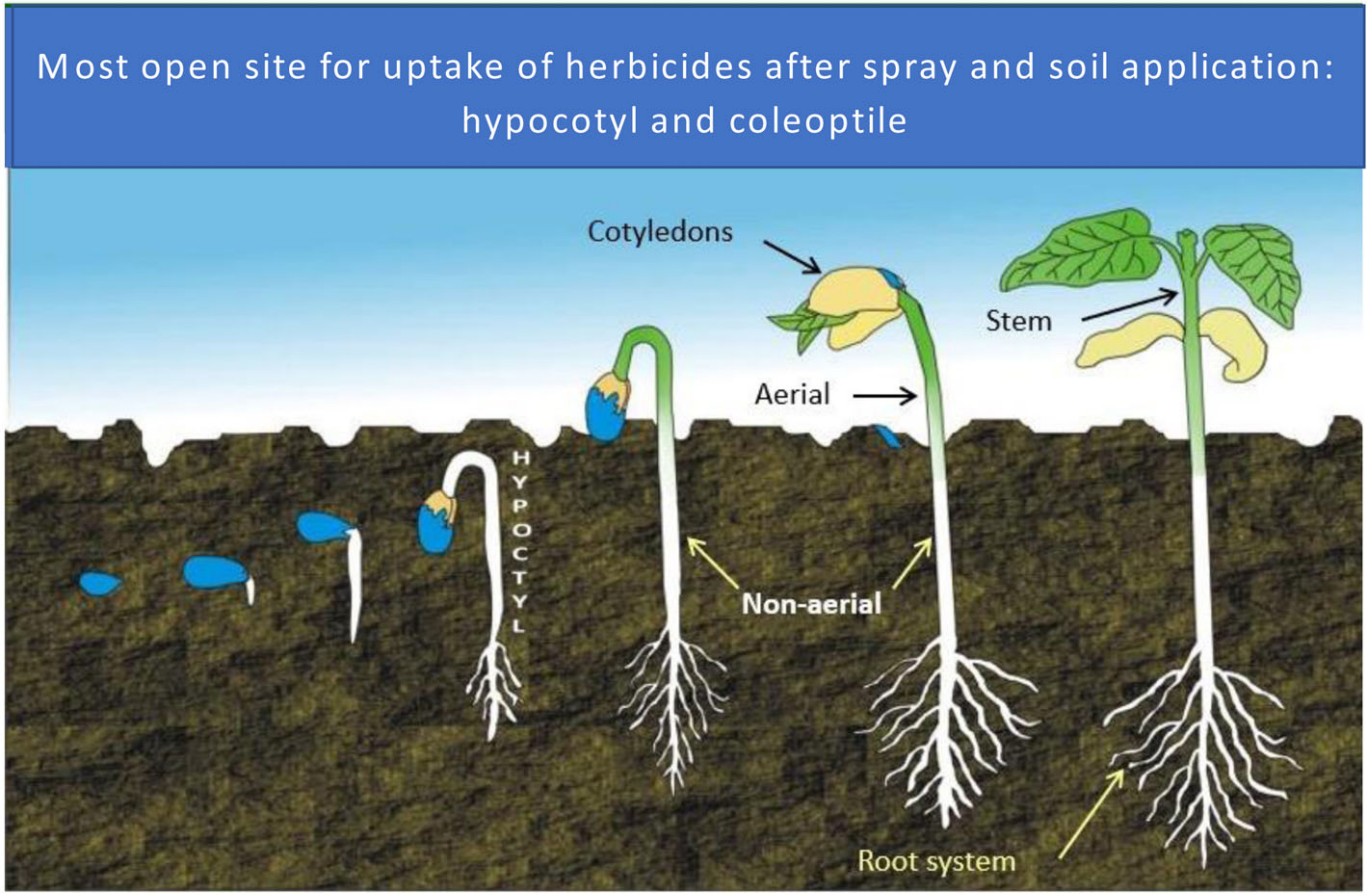
Uptake of pre‐emergence herbicides into different dicot plant stages (designed by Aponte and Baur^3^).

In a strict sense, pre‐emergence herbicides are applied prior to the emergence of the crop or weeds.^2^ Most weed management guidelines take, however, the emergence status of the crop as a reference point only. This means pre‐emergence herbicides are either applied directly to the soil or to young weed seedlings (Fig. 1). Many pre‐emergence herbicides can also be sprayed on or incorporated into the soil before planting. In any case, they act in most cases via the soil. We do not regard typical burn‐down herbicides applied before sowing as pre‐emergence herbicides, especially not those controlling emerged weeds after foliar treatment.

Pre‐emergence or soil‐applied herbicides play a major role in wide‐row crops where early competition of weeds may hamper crop yields. They are common weed management tools in crops such as maize, soybean, cotton and sugar cane in many parts of the world.

Another reason for their use is that farmers often face a heavy workload in the beginning of cropping seasons. They are forced to control weeds in different crops planted at around the same time such as maize and soybean in the United States. Pre‐emergence herbicides allow farmers to split their weed control management measures ideally across crops. They apply pre‐emergence herbicides primarily in maize and post‐emergence herbicides in soybeans later as soybeans tolerate some early weed competition better than maize. The wide range of soybean planting dates, row width in corn and herbicide resistance also contribute to this preference.

Finally, pre‐emergence herbicides provide tools for the avoidance of weed resistance risks as they often inhibit different target sites compared with post‐emergence products. All these properties make them widespread tools.

**Figure 2:**
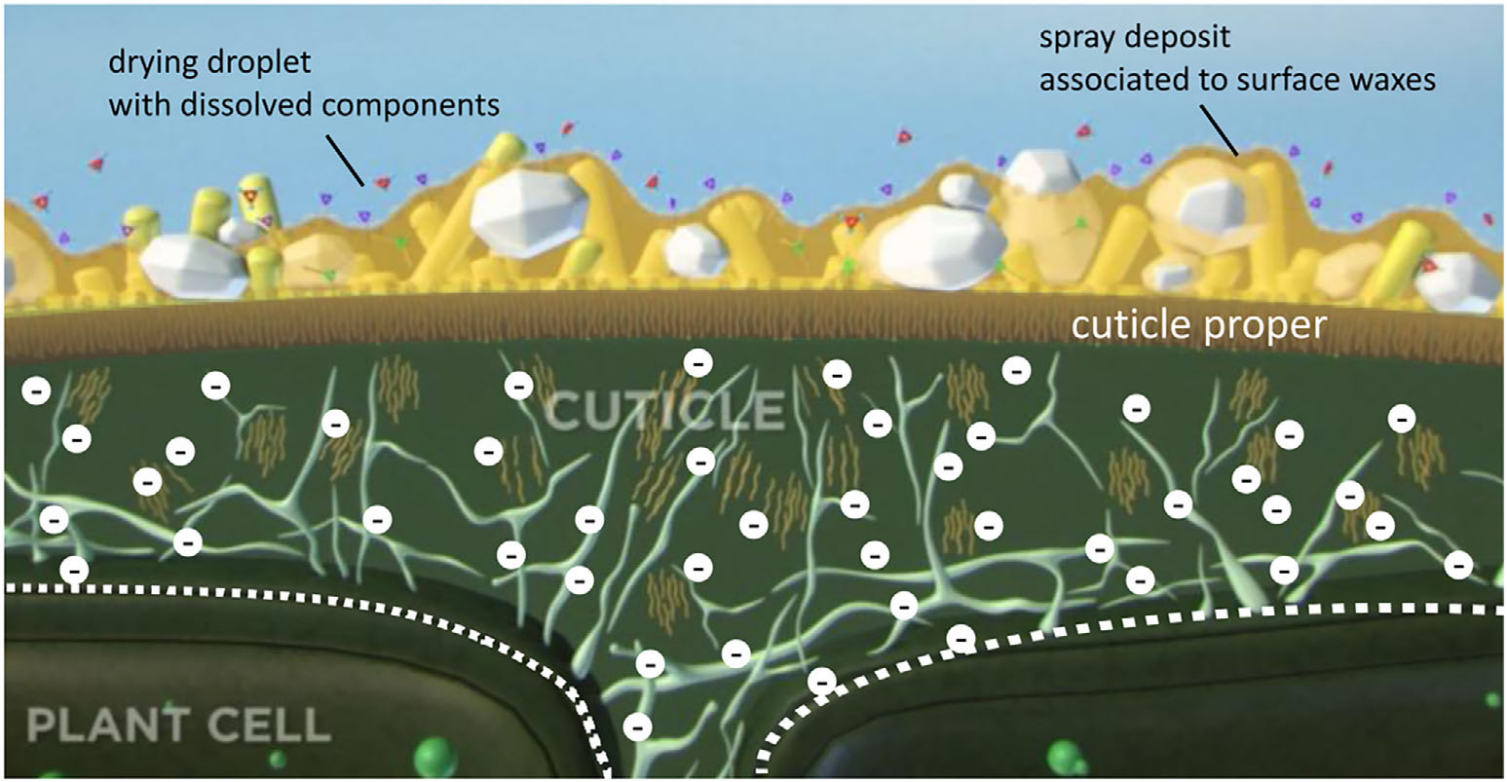
The cuticle is the first and main barrier to foliar herbicides. The cuticle proper is the real transport barrier. It is unrelated to surface wax (rods above cuticle proper) and not easily accessible by agrochemicals (white particles) and adjuvants (film above cuticle proper) or water respectively. White circles with a hyphen below indicate negative charges from carboxy groups which are particularly dense in the pectin layer (white dotted line) and limit transport of the ionized fraction of weak organic acid herbicides (modified from Aponte and Baur^32^).

Post‐emergence herbicides are sprayed on the foliage of crops and weeds. They either control escapees of pre‐emergence applications and/or they are applied several times for the control of sequential flushes of emerging weeds. An important barrier for post‐emergence herbicides is the leaf cuticle with waxes and very long‐chain fatty acid (VLCFA) derivatives (Fig. 2).

Herbicides are often distinguished by their mobility within plants. They are either classified as contact herbicides or as systemic herbicides.^2^ Immobile or rapidly damaging herbicides are classified as contact herbicides. Systemic herbicides translocate from sites of herbicide deposition to other parts of the plants and are therefore distributed throughout weeds. The general classification of compounds as contact herbicides can, however, be misleading and the term used should be amended by the term foliar applied, contact herbicides. Some compounds are not transported basipetally. They may, however, be taken up by roots and they may be transported acropetally leading to a distribution within the plant.

Pre‐ and post‐emergence herbicides are usually mixed with other herbicides as single components, and they usually do not control the full potential weed spectrum. For post‐emergence herbicides, timing is critical. In principle, however, most products perform best against young weed stages, usually in the two to four‐leaf stage. Later stages often require either higher dose rates or weeds are not sufficiently controlled. Weed management guidelines are provided by extension services in many countries around the globe. We mention here just two examples in the United States which highlight best timings for farmers in Michigan, Ohio, Indiana and Illinois.^4^, ^5^

Single herbicide applications for weed control will usually not provide sufficient weed control. Instead, sequential treatments of pre‐ and post‐emergence herbicides are common for the management of weeds germinating in flushes.

Herbicides are not developed by an academic process which allows the distinction between their pre‐ or post‐emergence characteristics. Companies want to recover their expenses and they can only develop molecules which can be registered. When scientists tried to find common physicochemical principles of marketed herbicides in the past based on physicochemical parameters, they often ignored this fact. A major factor for the selection of herbicides is their marketability and acceptance by the customer. They may have some properties in common but not as a result of their initial design as pre‐ or post‐emergence herbicides in the first instance.

Based on different sources, we estimate that the global market size for agrochemicals in the year 2019 was approximately 59 billion US$. The herbicide market that year amounted to approximately 30 billion US$. Neglecting all problems with an exact definition of the market for soil‐applied, pre‐emergence and pre‐plant herbicides and the possibility to use some compounds both pre‐ and post‐emergent and the special situation with rice herbicides, we assume a global turnover of soil‐applied herbicides at approximately 10 billion US$ and for post‐emergence products at 20 billion US$.

The growth of the post‐emergence segment in recent decades was driven by the use of glyphosate in transgenic crops (maize, soybean, cotton, sugarbeet, canola, and alfalfa). The overall increase of herbicide resistance has triggered the need to return to more complex weed management systems using a sequence of pre‐emergence followed by post‐emergence products, often used in combinations.

Some chemical classes are ideally suited for pre‐plant, pre‐plant incorporated, or pre‐emergence control. Examples are the chloroacetamides, thiocarbamates and dinitroanilines. The reason of their economic success over many decades has or had to do with the weed spectrum controlled, such as grass weeds in grass crops. Other compound classes are mostly or entirely post‐emergence compounds. Examples are acetyl‐coenzyme A carboxylase (ACCase) inhibitors such as aryloxyphenoxypropanoates and cyclohexanediones, auxins such as aryloxyalkanoic acid derivatives or the 5‐enolpyruvylshikimate‐3‐phophate synthase (EPSPS)‐inhibitor glyphosate. There are, however, compound classes as for example heterocycles in the group of hydroxyphenylpyruvate dioxygenase (HPPD)‐inhibitors from which both pre‐emergence and post‐emergence herbicides are in the market. Moreover, compounds from different compound classes such as atrazine, chlorsulfuron or mesotrione display pre‐ as well as post‐emergence activity at the same time.

This review addresses six different issues and objectives:

- Commercial aspects
  The development of herbicides is not driven by the directed design of herbicides as pre‐ or post‐emergence products. Companies have to recover their expenses. This means the potential performance of a compound in the market, whether pre‐ or post‐emergence or both, is the actual driving force.
- Regulatory aspects
  Physicochemical parameters have to be determined as regulatory requirements, primarily for the avoidance of environmental risks. Common properties between herbicides result more from this reason rather than from the application timing.
- Which physicochemical parameters favor the product uptake by the weed?
  Herbicides have to overcome different lipophilic plant barriers. As a consequence, one would assume that only herbicides with lipophilic properties can penetrate these barriers. Some herbicides are, however, quite hydrophilic.
- Which factors enable the transport of an herbicide to its site of action?
  Uptake is the first step of a compound to its target. It has to be translocated within the plant, also.
- Links between mode of action (MoA) and application timing
  Apparently, some compound classes are more suitable for soil application, others as foliar products. This fact can be correlated to some extent with some molecular MoAs.
- Synthetic approaches for the improvement of activity
  Chemists are interested in guidelines for chemical synthesis. We will try to answer whether simple physicochemical rules can improve the performance of soil or foliar herbicides.

## 2 COMMERCIAL ASPECTS

Costs for the development of new herbicide actives without crop tolerance traits amounted to approximately 340 million US$ in the year 2014.^6^ Today, we estimate that this figure is close to 420 million US$. Twelve to fourteen years are generally needed from the first synthesis to registration. This means that narrow spectrum, regional or specialty products cannot cover the investment accrued during their lifetime. New herbicides can only be developed in large acre crops such as wheat, maize, soybean or rice. These products have to fulfill all registration requirements of industrialized countries, and they have to show advantages over the state of the art herbicides against herbicide‐resistant weed biotypes. Costs at the farmer level should be in the range of existing weed management options.

## 3 PARAMETERS TO BE TESTED BY THE INDUSTRY EARLY ON

In parallel to early toxicity tests and to early environmental fate studies, potential groundwater leaching has to be evaluated with very early screening tests. In consequence, basic parameters are checked for active molecules at early screening stages: water solubility, p*K*_a_‐value, octanol–water partition coefficient (log *P*_oct_ or just log *P*), binding to soil particles (*K*_OC_) and dissipation half‐life time (DT_50_) in soil. An easily understandable overview regarding the role of different parameters in soil–herbicide interactions was compiled by Ross and Lembi.^7^

These factors are especially important for pre‐emergence herbicides, as these are applied early in the season when temperatures are often relatively low and when microbial soil degradation is delayed. When pre‐emergence compounds are applied, most of the active compound will hit bare soil. Model calculations with programs such as PEARL (https://www.pesticidemodels.eu/pearl/pearl-eu-leaching-soil-exposure) provide early indications of groundwater leaching risks.

Companies screening for herbicides have set early targets for the selection of compounds in order to exclude compounds with ground water leaching risks. Ideally, a new compound should show soil degradation half‐life times < 90 days, a log *P* < 2 and an effective applied dose rate below 200 g active ingredient (a.i.) ha^−1^.

Each factor can, however, influence the environmental fate of a compound. Lower dose rates and lower half‐life times will have positive effects. Each compound requires a specific analysis.

Preliminary data under laboratory conditions with one or two soil types will never be sufficient. Actual registration trials have to run with many soil types and in a variety of sophisticated tests. High vapor pressures/volatility are not desired from a registration and an application view.

As stressed earlier, weeds can germinate over a long time period. This is why residual activity of a pre‐emergence herbicide is desired by the farmer. However, the molecule must not have a too high half‐life time as this may result in a potential groundwater contamination or in a carry‐over and selectivity problem in the following crop.

Also, the pre‐emergence molecule has to reach underground seedling parts. In order to be adequately distributed in the soil – vertically and horizontally, some water solubility is required. Crop selectivity is sometimes dependent on positional effects and by the mobility of herbicides in soil. Roots of weed seedlings from a shallow depth may be affected, whereas crop seeds germinating from deeper soil areas are not. This kind of selectivity leads, however, to application restrictions, as, for example, is the case for metribuzin in sandy soils.^5^

As a result, all the regulatory requirements and the contrasting characteristics of a perfect soil herbicide make it quite difficult to develop a molecule for pre‐emergence applications. However, it is often possible to leverage a degree of placement selectivity with soil‐applied herbicides across a wider range of crops. Foliar herbicides, in contrast, are dependent on metabolic or target based selectivity which may be limited to specific crops only.

In general, the requested parameters for post‐emergence compounds are very similar to those of pre‐emergence herbicides. The probability of a post‐applied herbicide to hit bare soil is, however, much lower, especially in areas where the vegetation allows a fast development of crops and weeds. Temperatures in spring crops are usually higher when post‐emergence compounds are applied and favor soil metabolism and degradation of a chemical. These factors usually lead to reduced leaching risks. However, some residual soil activity is desirable for post‐emergence herbicides as well in order to control later weed flushes. Drift risks into adjacent sensitive crops and ecosystems may, however, be higher for post‐emergence products. Some of the earlier‐mentioned physicochemical parameters serve many purposes and also play a role in herbicide uptake models.

## 4 PLANT UPTAKE

The performance of an herbicide depends to a great extent on its uptake by weeds. Pre‐emergence herbicides are taken up by seeds before germination, by roots, by hypocotyls, by cotyledons (dicots), by the coleoptile (grasses) or by leaves (Fig. 1).

Trapp^8^ reviewed models describing the uptake of herbicides by roots. He described a few steps of herbicides on their way to their final target. He lists 16 parameters, including root volume, xylem surface, lipid fraction and others needed for the uptake‐calculation, independent from weed species. Using soybean as a model plant, he provides single, characteristic parameters. However, we believe that such uptake values can hardly be standardized, and that variability between individuals, growth stages and species have to be considered. Indeed, even Trapp found differences between barley and soybean. This fact made us analyze the uptake of an herbicide into a plant in a more detailed way, similar to Clarkson.^9^

**Figure 3:**
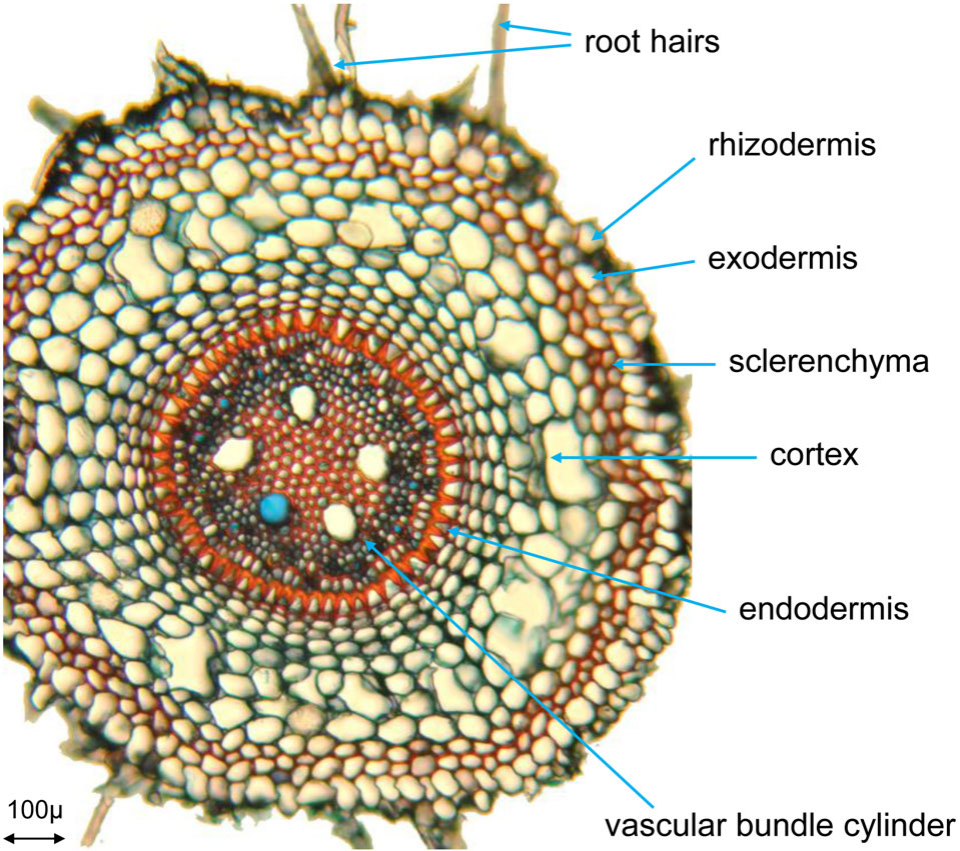
Transverse section of quackgrass (*Elymus repens* (L.) Gould) root (picture taken by H. Krähmer).

Compounds entering the root have to pass several cell layers before they can be transported by the vascular tissue (Fig. 3). The rhizodermis is the first layer. In many cases a special hypodermis, the exodermis, follows. Often, sclerenchyma layers confine the outer root cortex. The parenchymatic cortex part takes a considerable space then. And finally, the endodermis forms the border between the cortex and the vascular bundle cylinder. The rhizodermis of belowground roots usually does not develop a cuticle as the epidermis of aboveground organs do. Lignin is often formed in the walls of the rhizodermis. Waxes, cutin or suberin have shown to be more efficient barriers than lignin. Von Guttenberg^10^ reviews the literature on lipophilic incrustations into the rhizodermis which have a water‐repellent effect.

It is questionable from our point of view if lignin can prevent the apoplastic penetration of water and solutes into the root at all as herbicides are apparently easily transported through several layers with lignified cell walls as part of the apoplast.

The apoplast is the extracellular, supposedly dead part of the plant, and it includes the cell walls. The composition, the pH in the apoplast and the cellulose architecture stay, however, under the control of the symplast. Solutes can be transported in the apoplast, the symplast and along transmembrane pathways.^11^ The symplast is the living part of a plant and can be defined as the continuum of communicating cytoplasm. Water and solutes can enter the symplast from outside the plant via root hairs, and also at any place in the plant from the apoplast.

**Figure 4:**
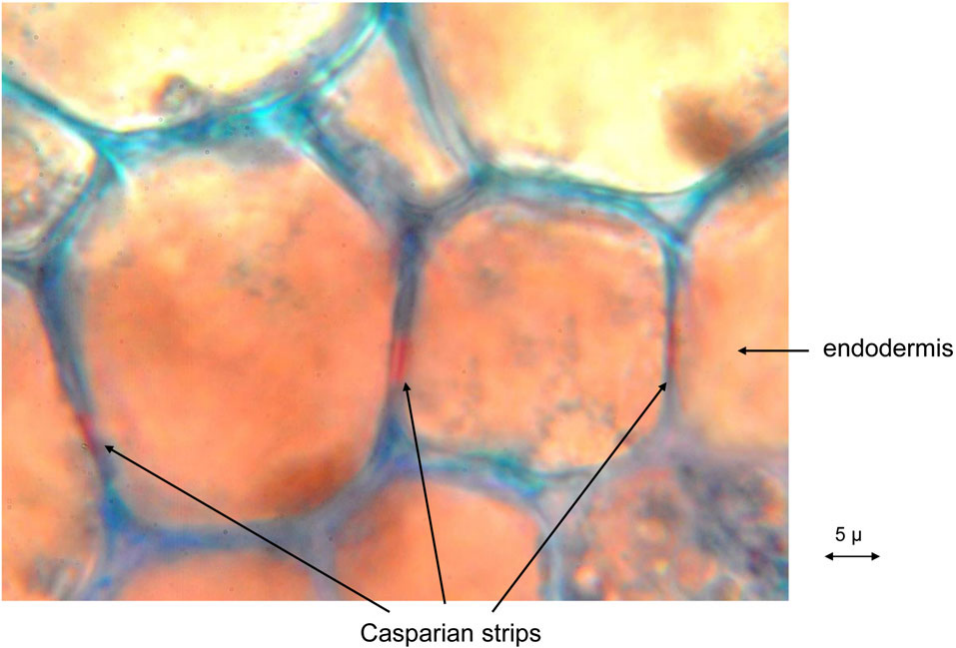
Horsetail (*Equisetum arvense* L.) rhizome with Casparian strips (transverse section) (picture taken by H. Krähmer).

In two cell layers of the cortex, cell wall modifications can be found which were assumed to control the apoplastic transport of water and solutes: the Casparian strip or band. Casparian strips also occur in the endodermis, at the border between cortex and vascular bundle cylinder of many weed rhizomes (Fig. 4).

**Figure 5:**
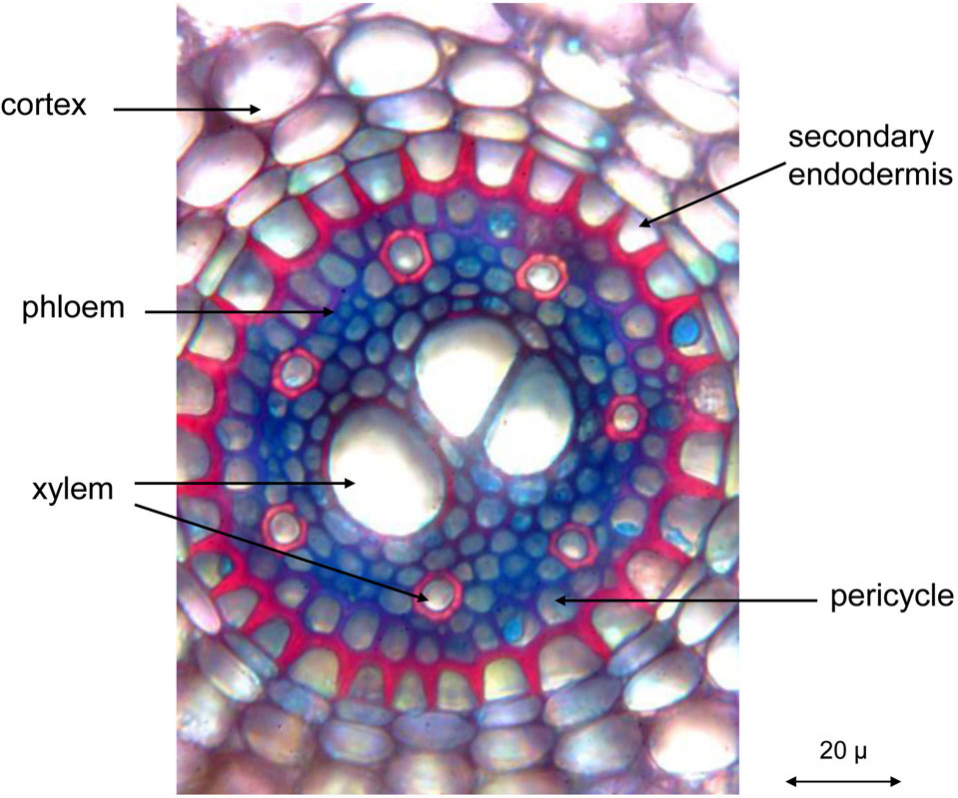
Quackgrass (*Elymus repens* (L.) Gould) root with secondary endodermis (transverse section) (picture taken by H. Krähmer).

Near the end of the last century, Perumalla *et al*.^12^ demonstrated that the exodermis of many species contains the Casparian strip in its anticlinal walls also. The endodermis, the innermost layer of the cortex^13^ is a sheath that has an impact on the transport of water and solutes into the central vascular bundle cylinder. The deposition of suberin lamellae on endodermis cell walls at later differentiation stages^13^ provides protection against the entry and loss of water as shown in the secondary endodermis rings of Figs 3 and 5. In many grass species, the thickening of the endodermis is unilateral and creates the impression of u‐shaped cell walls. Secondary and tertiary endodermis cells can, however, be surrounded completely by suberin containing layers also as for example in *Briza media* L. roots.^14^

### 4.1 How do all of these biological features relate to and influence the physicochemical parameters of herbicidal molecules

Root uptake was analyzed in the past with radiolabeled substances or with specifically designed trials guaranteeing root uptake only. Briggs and Bromilow^15^ tested a number of compounds and came up with a log *P* > 1.5 for the optimum uptake of compounds by barley roots. Unfortunately, this figure is often extrapolated into other plant species. It is obvious that root endodermis characteristics can differ considerably for different species.^16^

Despite the lipophilic character of the endodermis and the high lipophilicity of many pre‐emergence herbicides, it was documented many decades ago that highly water‐soluble compounds can also be taken up by roots. This was either directly demonstrated with radiolabeled material as in the case of picloram^17^ or indirectly with herbicidal effects after the application of herbicides to hydroponic culture as in the case of other auxinic herbicides.^18^ For further, highly water‐soluble herbicides, specific root bioassays were developed.^19^

Recent investigations of genes involved in the endodermis formation of *Arabidopsis thaliana* (L.) Heynh. mutants led to new insights into the composition of the endodermis. In the past, various components were claimed to form the Casparian strip as the major barrier for the translocation of xenobiotics into the root: suberin, lignin, lignin‐like polymers, or all of them. Naseer *et al*.^20^ proved that it is just a lignin polymer that forms the Casparian strip in *Arabidopsis*. It has to be shown if this is true for other species as well.

**Figure 6:**
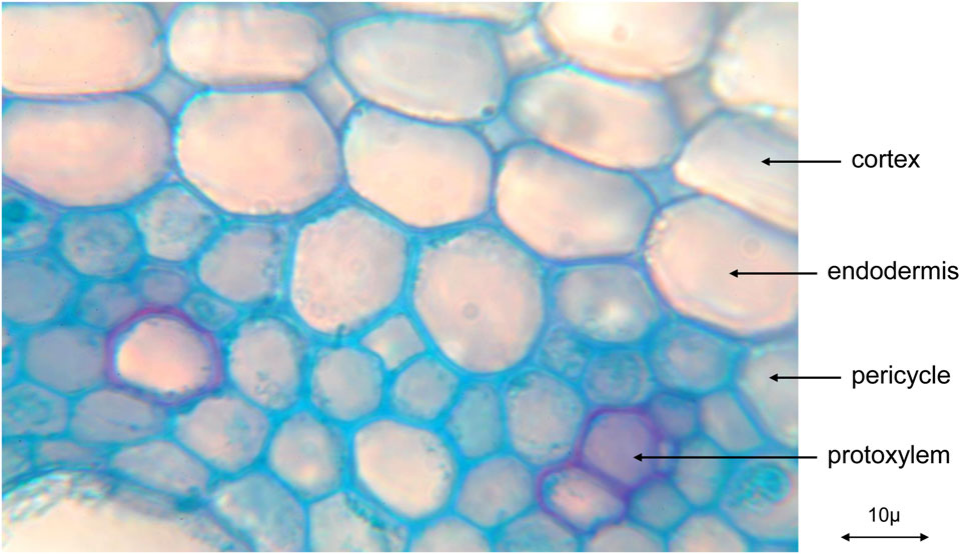
Young root of *Echinochloa crus‐galli* (L.) P. Beauv. without Casparian strip in the endodermis (transverse section) 70 mm from the root tip (picture taken by H. Krähmer).

The consistency of the endodermis has to be discussed in this context also. It differentiates with the age of roots from the tip to its base. Von Guttenberg^10^ claimed that the endodermis in its primary stage is not a real barrier to apoplastic water. The analysis of the differentiation of *Arabidopsis* roots improved our understanding of sequential developental stages.^21^ Root tips and the root hair zone are not protected in the same way as older root stages are. Some authors claim that Casparian strips or bands mature within 10 to 15 mm from the root tip.^22^, ^23^ Environmental conditions have an influence on the speed of maturation. Von Guttenberg^10^ reports the visibility of the Casparian strips around 5 mm of the root tip. In some cases, they can, however, not be detected by light microscopy up to 70 mm from the tip (Fig. 6). This is especially the case for roots of some species growing in water or moist soil. Water and solutes are primarily taken up by the root tip. Root hairs play a major role in this context.^10^ Modeling root uptake with log *P*‐values of known herbicides often leads to the assumption that lipophilic barriers prevent the apoplastic transport of water and xenobiotics as many soil herbicides are characterized by high log *P*‐values. Apoplastic, lipophilic barriers do, however, not exist at early growth stages of roots. And in fact, several highly hydrophilic herbicides are easily taken up by roots. Examples are several acetolactate synthase (ALS)‐inhibiting and auxinic herbicides. Even compounds which are regarded as ideal post‐emergence herbicides such as glyphosate^24^, ^25^ or glufosinate^26^, ^27^ are taken up by roots in hydroponic or water culture.

For acropetally translocated herbicides, the actual barrier from the apoplast to the symplast within stems is, however, the plasmalemma of cortical transfer cells next to the xylem.

These are quite frequent in stem nodal regions.^28^ In consequence, the root may not necessarily be the primary bottleneck we are looking for when analyzing the uptake of compounds by a plant.

As shown in the beginning of this section, compounds can also be taken up by the hypocotyl of dicots, by cotyledons or by early stem and leaf stages. In all these cases, an epidermis and parenchymatic cells have to be penetrated before a compound can enter the vascular bundle system.

Particularly striking and of paramount significance for soil‐applied herbicides is the non‐aerial and less so the aerial hypocotyl as shown for example for mung bean and canola and the coleoptile of grasses.^29^, ^30^ Penetration of herbicides into these organs is extremely high, sometimes close to quantitative after some hours, even without any enhancement by means of formulation or with adjuvants, and much faster than through any cuticle where polar routes have been claimed. In fact, these striking results on the openness of the hypocotyl suggest the lack of a cuticle at early plant developmental stages or the presence of pore like openings. Of course, the hypocotyl cuticle of fully differentiated plants may be rather thick and an effective barrier even for plant pathogens.^31^

Different methods were employed to understand the uptake process including osmotic measurements, studies on the impact of pH, staining with cationic and anionic dyes, radiolabeled tracer studies and here also measurement of the size selectivity of penetration.^3^, ^29^, ^30^, ^32^, ^33^ Independent of the method, the ‘cut‐off’ size of these pores is below 1.5 nm. While polyethylene glycol (PEG)200 to PEG600 rapidly penetrated in bulk amounts, PEG1000 with a radius in water of 0.85 nm could not enter. There is a strong pH dependence, suggesting repulsion exclusion of anionics. Cationic dyes like methylene blue stain strongly, but larger molecule size like astra blue do not stain at all, because they exceed the cut‐off size.

In monocots, the stem differentiates usually relatively late compared to dicots. The first monocot structures developing opposite the root are in some species the mesocotyl and in all species the coleoptile. The young stem of most monocots is enclosed by leaves for many days after germination. The uptake of soil herbicides by the coleoptile of monocots was demonstrated by selectively applied or by radiolabeled molecules in the last decades of the twentieth century. Jablonkai and Dutka^33^ showed that acetochlor is mainly taken up by roots of maize. The uptake by the coleoptile seems, however, to play a role for its selectivity as these authors demonstrated. One important factor that has to be considered when discussing soil performance is the interaction of herbicides with soil. Herbicides may be bound to soil particles,^34^ others have too short a half‐life time in soil for efficient weed control. Weeds can emerge in flushes and easily escape herbicides without residual activity. Consequently, sequential treatments with residual pre‐emergence herbicides followed by post‐emergence herbicides for the control of escapees or late germinating weeds have proven to be the most efficient weed control systems, as for example management programs involving glufosinate in soybeans.^35^

**Figure 7:**
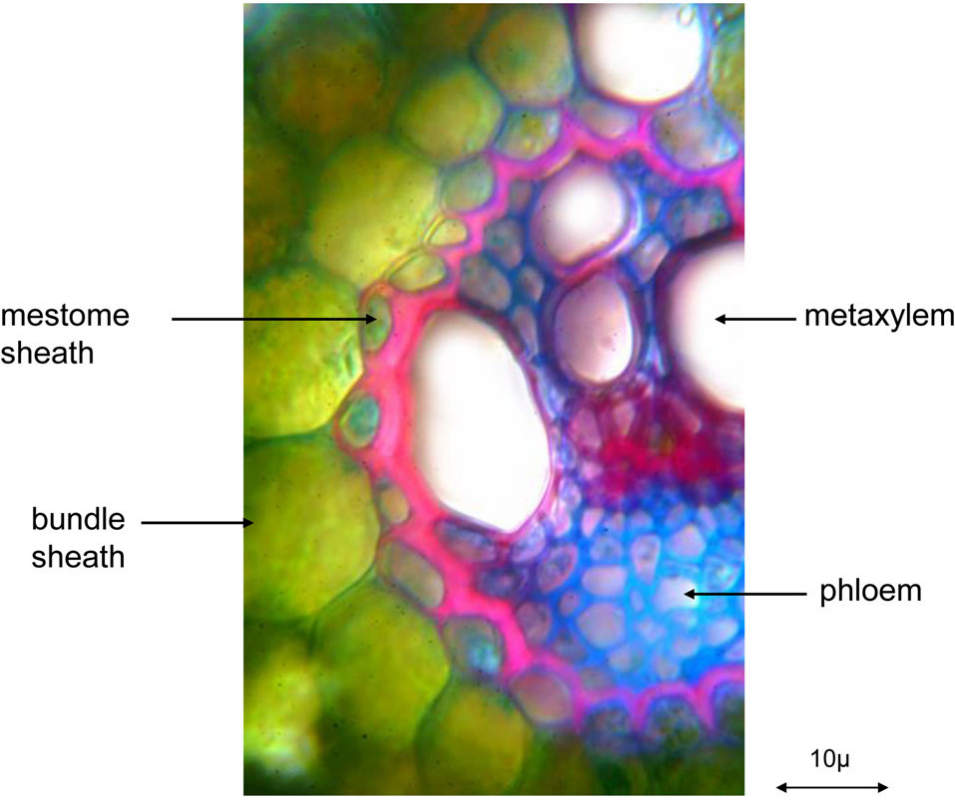
Leaf vascular bundle of blackgrass with mestome sheath as barrier to basipetally translocated herbicides (transverse section) (picture taken by H. Krähmer).

Post‐emergence herbicides enter plants primarily via leaves. The surface of plant organs is covered by structures which function as the first barriers to many xenobiotics (Fig. 2). The mestome sheath of vascular bundles is another plant border, especially for systemic post‐emergence herbicides. It is impregnated with suberin. Its structure (Fig. 7) is similar to that of the root endodermis. Lipophilicity seems therefore to be a prerequisite for the penetration of waxy and suberized plant barriers.

Some post‐emergence herbicides such as glyphosate and glufosinate are, however, quite hydrophilic. Compared to most other post‐emergence herbicides, glyphosate is not taken up very well, however, once taken up it is readily translocated and is very effective. Some authors claim that there are two paths through the cuticle, one for polar and one for lipophilic compounds.^36^ Aqueous pores were postulated by several authors in this context.^37^, ^38^

Formulation chemists have used isolated plant cuticles for penetration studies and have shown that these cuticles can be penetrated. Temperature and solute characteristics play a role when characterizing permeability.^39^

We will highlight now a few major aspects of foliar agrochemical penetration, for more detailed overview publications we refer for biology to Hull,^40^ for physicochemical aspects of pesticide behavior to Hartley and Graham‐Bryce,^41^ for cuticular penetration and the effects of adjuvants to Baur^42^ and for the spray applied fate of agrochemicals to Myung et a*l*.^43^

Foliar penetration via the cuticle is typically better with young leaves or fruits, or generally growing plants, at higher temperatures and very high relative humidity, for herbicides with low molecular weight, more linear structure, low melting point, and a high water‐solubility if at the same time molecular weight is low as well. Before cuticle absorption and migration of solutes can start, effective spray retention and coverage is needed particularly for difficult to wet crop and weed surfaces.^43^, ^44^, ^45^ This is not affected by herbicides and essentially dependent only on formulation and adjuvants and the spray application technology. The impact of plant surface can be significant. The relevant factors of influence are for example leaf shape and length, crystalline surface waxes, density of trichomes, presence of stomata and dust on the surface and they control at the same time the microclimate on the leaf.^46^

The surface or epicuticular waxes does not play a role for foliar penetration beyond the effective area that might be reduced due to diffusion impedance of crystals.^47^ The lack of significance as a barrier holds for both, amorphous and crystalline surface waxes and in fact the amorphous ones may ease absorption. Intracuticular, cutin‐associated waxes are defining the actual barrier properties.^42^

Highly relevant for good bioavailability of foliar applied herbicides is the molecular size of the solute as there is an exponential dependence of mobility in the cuticle on molar volume which itself follows for a given species and temperature in a log‐normal distribution.^47^

This exponential relationship was found over the entire range of foliar‐applied herbicides for all plant species studied, but the absolute permeability among species can vary by more than one order of magnitude for the speed of penetration.^37^ However, molecular size of herbicides causes differences in mobility of more than 100‐fold and the extremes are on the lower side molecules like amitrole (84.08 g mol^−1^) or dicamba (221.04 g mol^−1^) and the high molecular weight herbicides like tembotrione (440.8 g mol^−1^) or saflufenacil (500.85 g mol^−1^). The former small ones are very mobile and volatile and do not need penetration enhancers or other adjuvants, while the large molecules need methylated seed oil‐based tankmix adjuvants or other emulsifiers or suitable penetration enhancers to act at commercially reasonable use rates.^38^, ^43^

## 5 TRANSLOCATION

Penetration alone will, however, not necessarily guarantee the successful movement of the herbicide to its molecular target. As Caseley and Walker^48^ described decades ago, herbicides need to be transported to their site of action. Penetration and transport often require different characteristics. Lipophilicity will favor the uptake of a molecule. Translocation within the plant requires, however, some water solubility.

Entomologists and mycologists regard compounds taken up by the roots and transported by the xylem as systemic besides those transported in the phloem. This can be exemplified by the latest generation of fluorinated nicotinic acetylcholine receptor (*n*AChR) competitive modulators^51^ such as the insecticide sub‐groups (a.i.s) sulfoximines (sulfoxaflor),^50^ butenolides (flupyradifurone)^51^ and mesoionics (triflumezopyrim)^52^ demonstrating a strong acropetally xylem movement in plants. The new systemic fungicide oxathiapiprolin that targets an oxysterol‐binding protein is another example.^53^

Several pre‐emergence herbicides are taken up by roots and are transported acropetally within the xylem before they hit their targets. Examples are molecular targets within chloroplasts such as photosystem II (PS II), protoporphyrinogen oxidase (PPO) or any of several carotenoid biosynthesis enzymes, such as phytoene desaturase (PDS). In contrast, many post‐emergence herbicides have to be transported basipetally within phloem sieve tubes for the most efficient control of weeds. This is especially needed for herbcides to control perennial weeds which spread with underground propagules such as Johnsongrass (*Sorghum halepense*) or quackgrass (*Elymus repens*). Pre‐emergence products with acropetal translocation alone will usually not provide an acceptable control of perennial weeds. Most weed scientists do therefore not follow the systemicity definition of insecticide and fungicide scientists. For them, only basipetally translocated herbicides are true systemic herbicides.

Many studies have confirmed that phloem mobility can usually be achieved with weak acids. Mathematical models were developed in the past to calculate optimum conditions for the uptake and translocation of herbicides.^54^, ^55^ Physicochemical parameters for compound uptake and translocation can for example be linked. This approach results in contour plots as reviewed by Hsu and Kleier.^56^ One aspect that has been neglected in many models is the question of how many molecules have to arrive at the molecular target within a given timeframe to stop the growth of the target weed.

## 6 LINKS BETWEEN MODE/SITE OF ACTION AND APPLICATION TIMING

Table 1 shows different MoAs or targets of pre‐emergence herbicides with approximated log *P*‐values. Most pre‐emergence herbicides are rather lipophilic with log *P*‐values between 1 and 5.

**Table 1:** Modes of action (MoAs) favoring pre‐emergence treatments (data from EPA fact sheets, Pesticide Property Database, PubChem and different literature sources)

MoA, target site	Chemical family	Examples	Log *P*‐values
Microtubule organisation	Arylcarbamates and thiocarbamates	Propham, EPTC, chloropropham,	2–3
	Chloroaliphatic acids	TCA, Dalapon	1–2
Photosystem II‐ inhibitors	Pyridazines	Chloridazon	1
Very long‐chain fatty acid biosynthesis	Chloroacetamides	Allidochlor, alachlor, metolachlor	2–3
Microtubule assembly	Dinitroanilines	Trifluralin, pendimethalin	5
Cellulose biosynthesis	Nitriles	Dichlobenil	3
	Benzamides	Isoxaben	4
	Triazolocarboxamides	Flupoxam	5
	Alkylazines	Triaziflam, Indaziflam	3–4

A major question to be answered in this context is whether penetration through apoplastic plant barriers in the cell wall has to be considered first when discussing optimum physicochemical properties of herbicides or if in fact some membrane receptor sites such as PPO require lipophilic properties. In addition, we may ask ourselves, in which plant parts the inhibition of a given target site will cause the actual herbicidal effects. Photosynthesis‐inhibitors, carotenoid biosynthesis inhibitors and PPO‐inhibitors will only be effective in aboveground organs as the processes they inhibit require light. Some of these inhibitors with claimed pre‐emergence activity are just taken up by roots. They lead, however, to effects only when the plant emerges. Most commercially sold cellulose biosynthesis inhibitors primarily affect roots. The inhibition of cell plate formation caused by isoxaben in root tips was referred to by Dietrich et *al*.^57^ Light is clearly not required for their action. EPSPS is a plastid‐localized enzyme, but it is essential for metabolic processes in the absence of light.^58^ The same is true for the grass ACCase inhibited by aryloxyphenoxypropanoates and cyclohexanediones.^59^ Both groups of herbicides are typical post‐emergence herbicides. Only high dose rates of herbicides in these compound classes may lead to pre‐emergence effects.

Table 2 lists log *P* values for a few selected post‐emergence herbicides. They range between −5 and +5.8. From our point of view, it is impossible to conclude that post‐emergence herbicides can be characterized by their log *P*.

**Table 2:** Modes of action (MoAs) of some selected post‐emergence herbicides and approximated log *P*‐values (data from EPA fact sheets, Pesticide Property Database, PubChem and different literature sources)

MoA, target site	Chemical family	Examples	Log *P*‐values
Auxins	Aryloxyalkanoic acid derivatives	2,4‐D, fluroxypyr, 2,4‐D octyl	‐1 to 3 5.8
	Pyridinecarboxylates	Halauxifen‐methyl	3
Photosystem II‐ inhibitors	Biscarbamates	Phenmedipham	4
5‐Enolpyruvylshikimate‐3‐phophate synthase (EPSPS)	Glycines	Glyphosate	−5
Acetyl‐coenzyme A carboxylase	Aryloxyphenoxy‐ propanoates	Diclofop‐methyl fluazifop‐*p*‐butyl	5
	Cyclohexanediones	Alloxydim, Sethoxydim	3–4
Glutamine synthetase		Glufosinate	−5
Acetolactate synthase (ALS) (also known as acetohydroxy acid synthase, or AHAS)	Sulphonylureas	Metsulfuron‐ methyl	2

Table 3 shows compounds which exhibit pre‐ and post‐emergence activity to a considerable extent. Again, the log *P* values for the compounds listed in this table do not allow clear correlations with their pre‐ and post‐emergence efficacy.

**Table 3:** Modes of action (MoAs) of some selected herbicides with pre‐ and post‐emergence activity and approximated log *P*‐values (data from EPA fact sheets, Pesticide Property Database, PubChem and different literature sources)

MoA, target site	Chemical family	Examples	Log *P*‐values
Photosystem II‐ inhibitors	Arylureas	Monuron, diuron, isoproturon, linuron	3
	1,2,4‐Triazinones	Metribuzine	1.6
	1,3,5‐Triazines	Atrazine, simazine	2–3
Protoporphyrinogen oxidase	Diphenyl ethers	Nitrofen, acifluorfen, saflufenacil	2–4
	Oxadiazoles	Oxadiazon	5
Phytoene desaturase	Pyridazinones	Norflurazon, diflufenicane	2–5
Acetolactate synthase (ALS) (also known as acetohydroxy acid synthase, or AHAS)	Sulphonylureas	Chlorsulfuron, sulfosulfuron, pyroxsulam	−1 to 2
	Imidazolinones	Imazapyr, imazethapyr	1–2
Hydroxyphenylpyruvate dioxygenase‐inhibitors		Sulcotrione, mesotrione, topramezone	1–2

## 7 AGROCHEMICAL DESIGN AND SOLUTIONS IN CHEMISTRY BASED ON PHYSICOCHEMISTRY

A considerable number of herbicides are pro‐drugs or pro‐pesticides^60^, ^61^ which more easily penetrate the plant surface. They are converted into active metabolites within the plant. The converted biologically active herbicide is transported to the target site. Typical examples of post‐emergence pro‐herbicides are esters of some ACCase‐inhibitors. The esters are taken up by leaves faster than the free acids. They are metabolized by esterases in the plant.^62^

The same principle applies to various auxin herbicides applied as esters. Several pre‐emergence herbicides are also pro‐drugs such as thiocarbamates which inhibit the biosynthesis of VLCFA after sulfoxidation.^63^ Another example is the HPPD‐inhibitor isoxaflutole, an isoxazole which is converted into the diketonitrile.^64^

The knowledge of physicochemical properties of agrochemical products is helpful for the design and selection of libraries in combinatorial chemistry, high‐throughput (HT) and virtual screening technologies or for discovering novel agrochemical lead structures. The trend of physicochemical data of active ingredients has shown, that there is an increasing percentage of agrochemical products with higher hydrophobicity (log *P*‐values), and an increasing number of rotatable bonds. This may reflect the efforts of exploring hydrophobic interactions and different target‐binding structures for enhanced activities. Finally, by physicochemical profiling of launched products, the number and types of new lead structures in crop protection have significantly increased.^65^

However, by using independent and interpretable, molecular properties such as molecular weight, log *P*‐value, number of hydrogen bond acceptors and donors, number of rotatable bounds and aromatic rings a quantitative assessment has the ability to rank molecules whether they fail well‐established pesticide‐likeness rules or reach them and enable by this an efficient way for pesticide prioritization. These findings are of enormous value for an efficient estimation of pesticide‐likeness of large chemical libraries in the field of agrochemical research.^66^

For several years, the increased awareness of the organic solid state of agrochemical products and its physicochemical prospects led to changes in modern industrial workflows and the implementation of different types of study on solids. In this context, the knowledge of the thermodynamic stabilities and physicochemical properties of all potential solid forms support the design of agrochemical products. Their physicochemical properties, which show variations in terms of melting behavior, hardness, dissolution rate may influence the bioavailability of a polymorph, and can have a strong impact on the quality and efficacy of modern agrochemicals.^67^

## 8 DISCUSSION

Table 4 provides an overview on factors affecting the performance of soil and foliar applied herbicides. They make clear that uptake and translocation of an herbicide are not the only factors influencing its performance. Interactions of a chemical with soil can have a great impact on the efficacy of a soil‐applied herbicide.

**Table 4:** Parameters influencing the performance of soil and foliar applied herbicides

	Soil‐applied herbicide	Foliar applied herbicide
Soil texture/property	xxx	—
Soil pH	xxx	—
Influence of UV light	xxx	xxx
p*K*_a_	xx	xx
*K*_OC_	xxx	—
Log *P*	xxx	xxx
Water solubility	xxx	xx
Persistence	xxx	xx
Formulation	xx	xxx

Note: —, no or minor influence; x, small influence; xx, intermediate influence; xxx, highly influential.

Zhang et *al*.^68^ summarized a few property guidelines for the synthesis of modern pesticides. Their examples of post‐emergence herbicides listed in their review show huge differences for selected parameters. Log *K*_OW_ values range from almost −5 to nearly +6, p*K*_a_‐values from around 1.9 to 4.7. These differences are too high to allow immediate and sound correlations as we see it.

Passive uptake is not the only process by which compounds enter plants. Some herbicides are apparently taken up or exported by carriers as shown for glufosinate by Ullrich et a*l*.^69^

Also, the classification of compounds as systemic or contact by Zhang et *al*.^68^ should be disputed. Glufosinate‐ammonium is listed as a contact herbicide, despite existing publications on its translocation within plants.^70^ Some compounds such as glufosinate‐ammonium, chlorsulfuron and even glyphosate can limit their own transport as reported by Beriault et a*l*.^71^ and Geiger and Bestman,^72^ respectively. This fact should, however, not lead to their classification as contact herbicides. Mid‐day *versus* evening applications of these and other post‐emergence herbicides can also influence their translocation.

However, the PDS inhibitor diflufenican is sometimes defined as a contact herbicide^68^ based presumably on post‐emergence studies only. Bleaching symptoms in seedlings after pre‐emergence applications^73^ can, however, only be explained by an acropetal translocation.

Mathematical models trying to unify all parameters for the design of new molecules ignore metabolic events within plants and the fact that the actual active principle of an herbicide *in planta* may look completely different from that of the sprayed product. Keeping in mind that barriers in leaves and roots of weeds vary from species to species and differ depending on developmental stages, it appears quite difficult to develop general rules for the uptake of compounds in any plant species and for those reasons there are no ‘standard’ physicochemical parameters to differentiate between pre‐ and post‐emergence herbicides.

The title of our review may create wrong expectations. Synthesizing chemists might expect a few simple physicochemical rules as guidelines for their synthesis programs. Some tables or diagrams in scientific articles or textbooks sometimes create the impression of the availability of such tools. Our experience with several hundred thousand compounds tells us, however, that it is presumably not possible to turn pre‐emergence herbicides easily into post‐emergence herbicides and *vice versa* by modifying single parameters such as molecular weight, melting point, water solubility, lipophilicity or the introduction of functional groups. For decades, it was not possible to convert acetamides, dinitro‐anilines or thiocarbamates into real post‐emergence compounds nor EPSPS‐, glutamine‐synthetase nor ACCase‐inhibitors into pre‐emergence compounds as far as we know. Some MoAs might allow some flexibility for the design of the application timing such as in the case of ALS‐inhibitors or HPPD‐inhibitors. Principle instructions for the design of pre‐ or post‐emergence herbicides are, however, not known to us.

## 9 CONCLUSION

The design of pre‐ and post‐emergence herbicides involves a multifactorial approach. Registrability and marketability as prerequisites have to be considered first. The optimization of efficacy can be steered by different physicochemical parameters, by the interactions of a molecule with the environment such as soil in the case of pre‐emergence herbicides, of ultraviolet (UV) light stability *versus* penetration in the case of post‐emergence herbicides and plant metabolism *versus in planta* stability. The high diversity of plant barriers for xenobiotics requires models derived from different plant species. Many herbicides are converted to the active principles within the plant. In consequence, optimization models have to consider a biphasic approximation to an ideal compilation of data. A considerable number of herbicides show both, pre‐ as well as post‐emergence activity.
